# Adding virtual plants leads to higher cognitive performance and psychological well-being in virtual reality

**DOI:** 10.1038/s41598-023-34718-3

**Published:** 2023-05-17

**Authors:** Fariba Mostajeran, Frank Steinicke, Sarah Reinhart, Wolfgang Stuerzlinger, Bernhard E. Riecke, Simone Kühn

**Affiliations:** 1 Human-Computer Interaction Group, Department of Informatics, Universitāt Hamburg, 22527 Hamburg, Germany; 2 VVISE Lab, School of Interactive Arts and Technology, Simon Fraser University, Surrey, BC V3T 0A3 Canada; 3 iSpace Lab, School of Interactive Arts and Technology, Simon Fraser University, Surrey, BC V3T 0A3 Canada; 4 Neural Plasticity Group, Clinic and Polyclinic for Psychiatry and Psychotherapy, University Medical Center Hamburg-Eppendorf, 20246 Hamburg, Germany; 5 Lise Meitner Group for Environmental Neuroscience, Max Planck Institute for Human Development, 14195 Berlin, Germany; 6 Max Planck-UCL Center for Computational Psychiatry and Ageing Research, 14159 Berlin, Germany

**Keywords:** Psychology, Computer science

## Abstract

Previous research has shown the positive effects of exposure to real and virtual nature. To investigate how such benefits might generalize to ever-more-prevalent virtual workplaces, we examined the effects of the absence or presence of virtual plants in an office environment in Virtual Reality (VR) on users’ cognitive performance and psychological well-being. The results of our user study with 39 participants show that in the presence of virtual plants, participants performed significantly better in both short-term memory and creativity tasks. Furthermore, they reported higher psychological well-being scores, including positive affect and attentive coping, whilst reporting lower feelings of anger and aggression after exposure to virtual plants in VR. The virtual office with plants was also perceived as more restorative and induced a higher sense of presence. Overall, these results highlight how the presence of virtual plants in VR can have positive influences on users, and therefore, constitute important design considerations when developing future working and learning spaces.

## Introduction

As a result of urbanization, more than 50% of the world’s population lives in urban areas, a proportion that is expected to further increase to 68% by 2050^1^. Already today, many people have only limited access to nature as artificially designed living spaces such as working environments often separate them from regular contact with nature. This limited access to nature has been further challenged during the COVID-19 pandemic, for instance, during phases of lockdown as well as numerous hours of teleworking, which might even be further extended in the future by novel forms of remote immersive working spaces and visions of the metaverse^2–5^.

In contrast, there is a vast body of literature that identifies evidence for the positive effects of nature on human’s mental and physical health^6^. For instance, exposure to nature has been shown to be effective in reducing physiological arousal markers such as blood pressure^7^, the stress-related cortisol hormone^8^, as well as self-reported feelings of aggression^9^ and anxiety^10^. Also, mood^11,12^ and psychological well-being^13^ have shown improvements as a result of interaction with nature.

The Stress Reduction Theory (SRT) provides a possible explanation for this effect^7^. According to this theory, natural environments promote stress recovery by normalizing physiological arousal responses, enhancing positive emotions, and reducing negative or stress-related feelings^7,14^. Since the biophilia hypothesis^15,16^ suggests that evolution may have equipped humankind with an innate connection to nature, natural environments tend to be significantly more restorative compared to the current artificial urban ones. Also, natural environments activate human’s parasympathetic nervous system in a way that reduces stress^7,14^.

Another outcome of interaction with and exposure to nature is a positive effect on cognitive abilities and functions^17^. Cognitive functions refer to several mental abilities which can be characterized by divergent and convergent cognitive processes^18^. Convergent cognitive processes are mostly related to intelligence and could be divided into the subcategories of attentional and data processing tasks, while divergent cognitive functions are related to creativity^19^. However, these cognitive resources or abilities are not infinite and may become fatigued^20^. For instance, voluntary allocation of attention to certain features, objects, or regions in space may result in fatigue, due to the focus on a task with little or no intrinsically motivational draw while simultaneously having to suppress more interesting input^21,22^. Kaplan and Kaplan^21^ argue in their attention restoration theory (ART) that natural environments are ideal places for humans to restore their diminished attentional capacity. The argument here is that natural surroundings capture our attention in a bottom-up (i.e., stimulus-driven) fashion, which allows our top-down (i.e., goal-oriented) directed-attention abilities to be restored^21,23^.

Even in virtual environments, similar positive effects of simulations of nature using immersive virtual reality (VR) technology have been observed^24,25^. For instance, as a result of exposure to immersive virtual nature previous studies have reported improvements in mood^26,27^ and cognitive performance^28,29^, recovery from stress^30^, as well as reductions of anxiety^31,32^ and negative emotions such as fatigue and depression^33^.

These findings are particularly crucial for urban environments where access to nature is limited. Therefore, architects, urban planners, and developers have identified a need to integrate more natural elements into different parts of buildings^34^. Biophilic design aims to create a living space for humans through different design strategies^35^. It incorporates natural elements such as natural materials, plants, views, and vistas into the indoor environment^36^. Other basic elements of a biophilic design include natural shapes, forms, and patterns as well as natural light and spatial harmony. Additionally, place-based relationships such as the geographic connection to a place and evolved human-nature relationships such as prospect and refuge play a role in such a design.

Specifically within the work context the positive effects of biophilic design have been demonstrated in real-world scenarios. For instance, Lohr et al.^37^ showed that plants in a windowless workplace corresponded to a 12% faster response time on a computer task. Similarly, Nieuwenhuis et al.^38^ found that a plant-enriched office increased productivity by 15%. Further, it has been suggested that creativity can benefit from exposure to nature. For instance, Shibata and Suzuki^39^ showed that the creative performance of women in an association task was significantly higher when there were plants in the room.

These findings have been partly confirmed in virtual settings as well. In a series of studies, Yin et al.^19,40,41^ examined the effects of immersive biophilic office designs on psycho- and physiological responses. In their first study^40^, participants were physically or virtually (through an immersive video of the same physical environment in VR) exposed to an indoor environment with or without plants. They observed that systolic blood pressure (i.e., the pressure in arteries when the heart beats) was significantly lower in both real and virtual biophilic environments compared to non-biophilic ones. However, diastolic blood pressure (i.e., the pressure in arteries when the heart rests between beats) as well as skin conductance levels were only significantly lower in the virtual biophilic environment compared to a non-biophilic one. These measures were not significantly different between biophilic and non-biophilic real environments.

In addition, three cognitive tests were administered and only one test (i.e., a visual backward digit span test) revealed a significant difference between conditions, with the real biophilic environment resulting in better cognitive performance (i.e., higher mean digit span) compared to a non-biophilic real environment.

In their follow-up studies, Yin et al.^19,41^ used computer-generated office environments in VR. Similar to an immersive video of a biophilic indoor environment, physiological indicators of stress such as skin conductance level showed consistently lower levels compared to non-biophilic virtual environments. In their 2019 study^19^, biophilic design showed positive effects on creativity whilst demonstrating a negative effect on convergent cognitive processes, i.e., longer reaction times when performing a Stroop test^19^.

Therefore, the effects of exposure to an immersive biophilic indoor environment, such as an office environment, on divergent and convergent cognitive processes as well as psychological well-being is not clearly understood. Furthermore, the biophilic designs used in Yin et al.^19,41^ were implemented by using a vast amount of natural artifacts including numerous plants, outside garden views, and vertical plant walls, which covered almost the entire room. This makes it difficult to attribute the observed effects to the specific design elements. Moreover, such comprehensive biophilic designs are infeasible in many situations, and it remains open how more minimalistic biophilic designs, which for instance introduce only a few plants in an office environment, affect a user’s psychological well-being and cognition. This motivated us to investigate if the presence of limited indoor biophilic features - virtual plants - in immersive VR would be sufficient to observe improved psychological well-being and cognitive functions compared to the absence of such attributes, a research question which remains largely unexplored.

Taking the biophilia hypothesis, stress reduction theory, and attention restoration theory into consideration, it is reasonable to hypothesize that the presence of virtual plants in a virtual office environment could have similar effects on human cognition and psychological well-being as in the real world. Hence, we hypothesized that **(H1)** compared to an exposure to the same virtual environment without plants, exposure to a virtual office environment with virtual plants leads to higher cognitive performance in **(H1.a)** a short-term memory task measuring convergent cognitive functioning and **(H1.b)** a creativity task measuring divergent cognitive functioning. In addition, we hypothesized that **(H2)** the presence of virtual plants in the virtual office environment leads to higher psychological well-being compared to the absence of plants. We also expected to observe **(H3)** higher perceived restorativeness ratings for our biophilic virtual office environment compared to a non-biophilic office environment. Finally, we hypothesized that **(H4)** the presence of virtual plants leads to a higher sense of presence in the virtual office environment.

## Methods

To test our hypotheses, we designed a within-subject study where the same virtual office environment was utilized either with or without virtual plants, and where participants (N = 39) performed a short-term memory and a creativity task while being in these virtual environments. In addition to these cognitive tests, participants rated their mood and feelings in self-reported questionnaires after exposure to each of the two environmental conditions on a separate computer outside of VR. In contrast to previous work in this area, we examined our independent variable in isolation, i.e., the only difference between the two virtual environments were the added virtual plants, which ensured that the observed effects cannot be attributed to other confounding variables.

### Virtual environments

**Figure 1:**
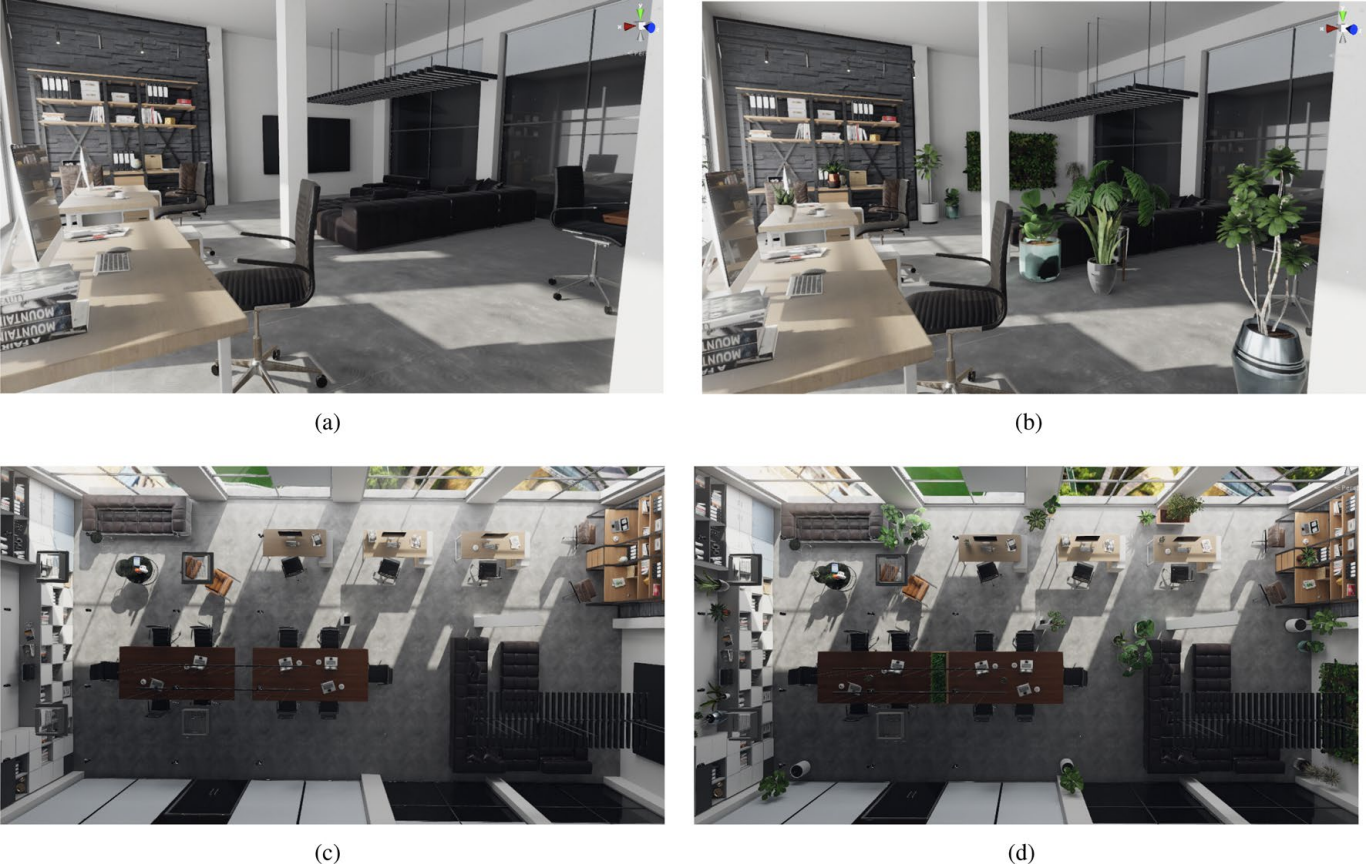
Example illustrations of the virtual office (**a**, **c**) without and (**b**, **d**) with virtual biophilic design in forms of virtual plants from the participant’s perspective in the experiment (**a**, **b**) and from a top view (**c**, **d**).

For this study, we modeled a virtual office environment (as illustrated in Fig. 1) with two different conditions:

- Condition “no-plants”: In this condition the virtual office was devoid of any plants (cf. Fig. 1a,c).
- Condition “plants”: In this condition the same virtual office was enriched by 28 virtual 3D models of plants, which were distributed as illustrated in Fig. 1b,d, to achieve an overall plausible office appearance.

We made use of several 3D models and materials to build our virtual office environment using the Unity game engine (version 2019.2.12f1), with the Unity asset Office Interior Archviz2 serving as our base virtual environment. Also, realistic lighting was generated using the Unity asset Bakery GPU Lightmapper3. In addition, for the office environment with plants, we placed 28 plants from different assets with additional manual modeling and a wall garden.

The virtual environments were displayed within an HTC Vive Pro (resolution 1140 $\times$ 1600 pixels; refresh rate 90 Hz) head-mounted display (HMD) with integrated headphones and controllers. It has to be mentioned that although the virtual environments did not have an ambient sound, we used the HMD’s integrated headphones to present our auditory cognitive tasks to the participants. To render the virtual environments, we employed a Windows 10 computer (NVIDIA Quadro RTX 8000, Intel Core i7 4790K with 4 GHz). Finally, a second Windows 10 computer (NVIDIA GTX 2080Ti, Intel Core i7 4790K with 4GHz) and its monitor (24”, resolution 1920 $\times$ 1080 pixels; refresh rate 60Hz) were used for filling out the questionnaires.

### Measures

We employed the following tests and questionnaires to evaluate cognition and psychological well-being in this study. These measures are widely used in the literature on real and virtual nature experiences^42^. This experiment was conducted in the local language. Therefore, all task instructions and questionnaires were given in the German language. A supplementary video shows the conduction of the cognitive tasks in the virtual office with plants in VR.

#### Digit span backward test (DSB)

As a measure of participants’ convergent cognitive functioning we used an auditory Digit Span Backward test (DSB)^43^, to assess the capacity of their working memory. In this task, participants heard a pre-recorded sequence of numbers via the VR HMD, which they had to repeat in reverse order. Initially, the sequence of presented numbers had a length of two digits. With each correct answer, the sequence of numbers was increased by one digit. For each wrong answer, participants received the previously presented length again. For two consecutive wrong answers, the sequence of numbers was reduced by one digit. The end of this task was reached after 14 trials. The evaluation of the DSB test is based on the analysis of the verbally repeated number sequences of the participants. The longest correctly answered sequence of numbers within the 14 trials was recorded as the maximum length (ML). In addition, we determined the mean span (MS) metric using the method described by Woods et al.^44^, as it has shown clear advantages, such as reduced variance, improved test-retest reliability, and higher correlations with the results of other neuropsychological tests, compared to other traditional digit span measures.

#### Alternative uses task (AUT)

The Alternative Uses Task (AUT) is a validated test for assessing creativity, which has been classified as a divergent cognitive process^45,46^. In this task, users have to list as many alternative uses as possible for an everyday object. We administered this task within VR and used pre-recorded audio for giving the instructions to the participants. The objects used in our experiment were (i) car tires, (ii) tin can, (iii) newspaper, and (iv) brick. We fully randomized the order of these objects for the experiment. Each participant received one of these orders during the experiment. The first two objects in the order were given during the first condition they received and the second two objects were given during the second condition. Therefore, none of the objects could be repeated across conditions for one participant. For each object they had two minutes time where they had to name as many alternative uses as they could think of. For the evaluation of the AUT, we used a paraphrasing transcription of the answers based on the audio files. Individual responses were then assessed for each object by five independent judges using snapshot assessment^47^. The judges were unaware of the condition the replies were generated in. This method aggregates the participant’s responses per item into a single, holistic assessment. The rating scale ranged from 1 (Not creative at all) to 5 (Very creative). In order to reach a coherent evaluation between judges, they were trained on Guilford’s suggested concepts of fluency (number of interpretable, meaningful answers), flexibility (number of different categories), originality (rarity degree of answers), and elaboration (degree of detail given per answer)^46^. The participants’ answers were presented to each judge for each object. For the analyses, we determined the average snapshot rating for each object by each individual rater. Then, these average ratings were added for the objects that were presented to the participants in each condition.

#### Positive and negative affect schedule (PANAS)

To measure the participants’ affective states, a part of their psychological well-being^48^, we used the Positive and Negative Affect Schedule (PANAS)^49,50^. It uses 20 adjectives to assess one’s current affective states. Ten of these adjectives measure positive affect (PA, e.g., interested, attentive), whereas the remaining ten items measure negative affect (NA, e.g., guilty, anxious). Participants rated each item on a five-point Likert scale ranging from 1 (Very slightly/not at all) to 5 (Extremely).

#### Zuckerman inventory of personal reactions (ZIPERS)

We further measured the participants’ affective responses to the VR exposure using the Zuckerman Inventory of Personal Reactions (ZIPERS)^51^. The ZIPERS breaks down a person’s feelings based on five factors: (i) Fear Arousal (FA), (ii) Positive Affect (PA), (iii) Anger and Aggression (Agg), (iv) Attentive Coping (Cop), and (v) Sadness (Sad). Again, participants rated the items on a five-point Likert scale ranging from 1 (Not at all) to 5 (Very much).

#### Perceived restorativeness scale (PRS)

This questionnaire measures perceived restorativeness of an environment, which is relevant to query different aspects of attention restoration theory^52–55^. The 26 items form the sub-scales (i) being away (BA), (ii) coherence (COH), (iii) compatibility (COM), (iv) fascination (FA), (v) scope (SCO), (vi) familiarity (FAM), and (vii) preference (PREF). Each item is rated on a seven-point Likert scale from 1 (Not at all) to 7 (Completely). In addition to the individual sub-scales, an overall value can be calculated for the analysis, which is defined by the average of the sub-scales BA, FA, COM, COH, and SCO.

#### Sense of presence

An essential feature of VR is its ability to evoke a sense of presence, i.e., a sense of being physically present in the virtual environment^56^. This characteristic leads to human behavior that is similar to the behavior shown in real environments. To measure the sense of presence in the virtual environments of this study, we employed the first item of the Igroup Presence Questionnaire (IPQ)^57^. This item was originally developed by Slater and Usoh in 1994^56^ and evaluates the general feeling of being in a virtual environment (“sense of being there”).

#### Simulator sickness questionnaire (SSQ)

During or after using VR, users may experience a syndrome similar to motion sickness with symptoms such as nausea, headaches, or dizziness. This syndrome is known as simulator sickness or cybersickness^58^. Although the biological causes of simulator sickness have not been confirmed yet^59^, several theories have tried to explain the responsible factors for experiencing simulator sickness. The most common theory is Sensory Conflict^60^ which explains that symptoms of simulator sickness will occur if the stimulus from the outside environment is being perceived differently by different senses of the user. We used the Simulator Sickness Questionnaire (SSQ) by Kennedy et al.^61^ to determine whether the entire experiment caused any simulator sickness symptoms. The SSQ uses 16 items to describe physical symptoms that can occur during or after exposure to VR, e.g., general malaise. The items are rated on a four-point scale ranging from 0 (Not at all) to 3 (Very much). The 16 items result in the three sub-scales for (i) nausea (NAU), (ii) oculomotor issues (OCU), and (iii) disorientation (DIS). Finally, summing all sub-scales multiplied by 3.74 calculates a total SSQ score.

### Participants

The study was approved by the local psychological ethics committee of the Center for Psychosocial Medicine at the University Medical Center Hamburg-Eppendorf and performed in accordance with relevant guidelines and regulations. Participants were recruited via an e-mail distribution list of the Department of Computer Science at the University of Hamburg. In addition, we advertised for the experiment via our social media channels. Inclusion criteria were that participants were at least 18 years old and did not suffer from any known health conditions. A total of 40 participants took part in the experiment. However, the collected data of one participant had to be excluded from the analysis due to technical problems right after filling out the demographic and SSQ questionnaires but before exposure to any virtual environment. The remaining 39 participants (23 women) were aged between 19 and 56 years ($M= 24.15, \textit{SD} = 6.03$) and all completed the experiment. However, one participant misunderstood the instructions of the creativity task (i.e., AUT), so that their responses could not be taken into account in the analysis. Further, as the microphone failed to record the responses of three participants, the data for the cognitive and creativity tasks were analysed only for the available responses (i.e., $N=36$ for DSB and $N=35$ for AUT).

### Procedure

The study was conducted in an ${\sim }60$ $\textrm{m}^2$ laboratory room at the Department of Computer Science at the University of Hamburg. Upon arrival in the lab, participants were welcomed and presented with the participant information and the data protection declaration forms. They were informed that the study investigates how being in virtual offices affects cognition and psychological well-being. Yet, they did not receive any information about our hypotheses and the fact that one of the office environments would contain plants whereas the other did not. After signing the informed consent, participants filled out demographic and SSQ questionnaires and put on the VR HMD. Then, they saw either the virtual office environment with or without plants, in randomized order. In the beginning, they had one minute time to explore the virtual office using the teleportation technique. After that, they were teleported automatically to a predefined location in front of a virtual sofa. Then, they were instructed to take a seat in the corner of the physical sofa in the laboratory room which was registered with, i.e., located at the same place as, the virtual sofa in the virtual office environment. During these tasks, teleportation was deactivated so that the participants could no longer leave their assigned place. The perspective of participants sitting on the sofa for both conditions is shown in Fig. 1a,b.

Once seated, participants listened to the AUT’s instructions and completed the task. After a short break, the DSB task followed. Subsequently, participants took off the HMD and filled out the questionnaires, i.e., PANAS, ZIPERS, PRS, and sense of presence. This procedure was repeated for the second condition. After experiencing both conditions, participants filled out the SSQ once more and answered some questions about the presence of real plants in their living and working environments as well as their experience in both conditions of this experiment. After that, they were compensated with course credits, if required. Exposure to each virtual environment in VR lasted for 12 minutes and the total experiment duration was about 50 minutes.

## Results

**Figure 2:**
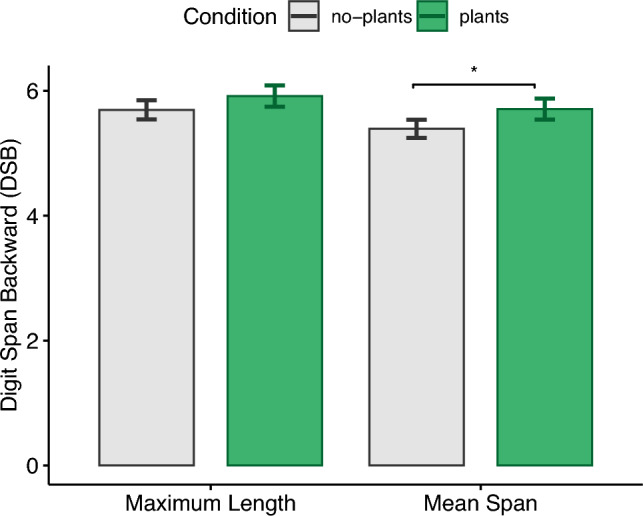
Digit span backward (DSB).

**Figure 3:**
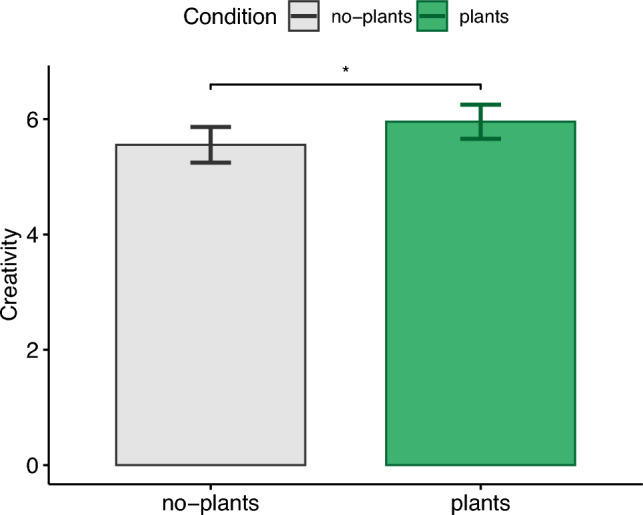
Alternative uses task (AUT).

**Figure 4:**
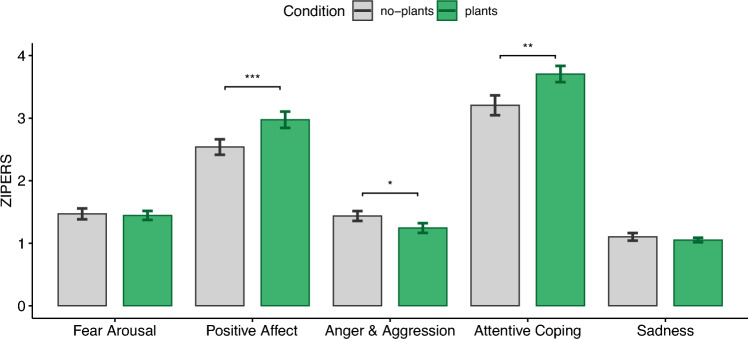
Zuckerman inventory of personal reactions (ZIPERS).

**Figure 5:**
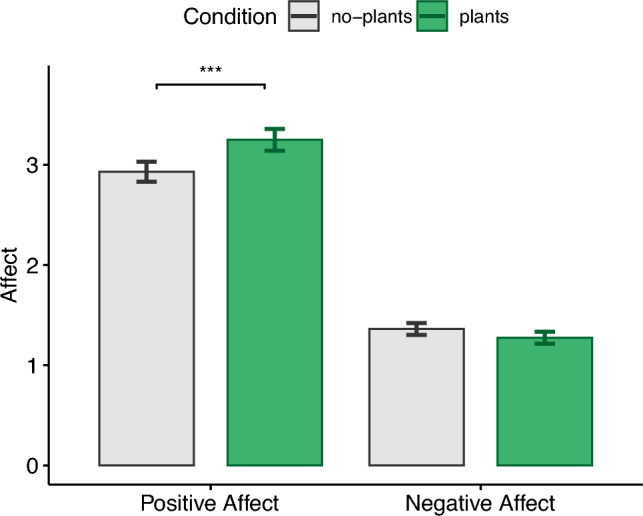
Positive and negative affect schedule (PANAS).

**Figure 6:**
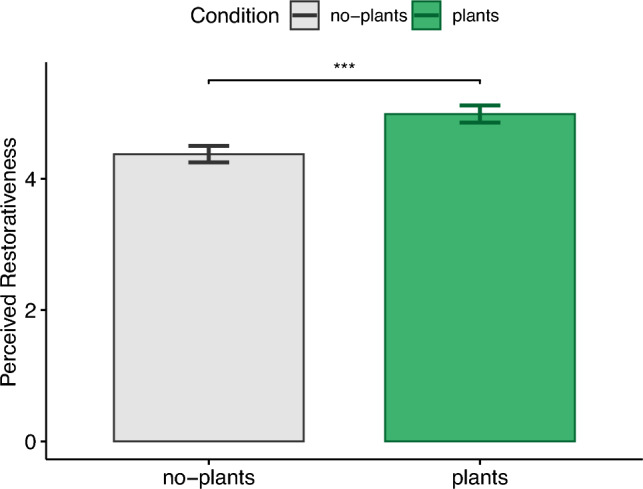
Perceived restorativeness scale (PRS) total score.

**Figure 7:**
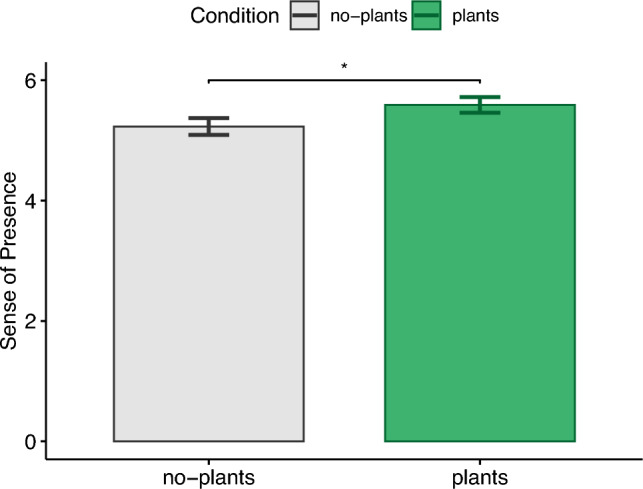
Sense of presence.

**Figure 8:**
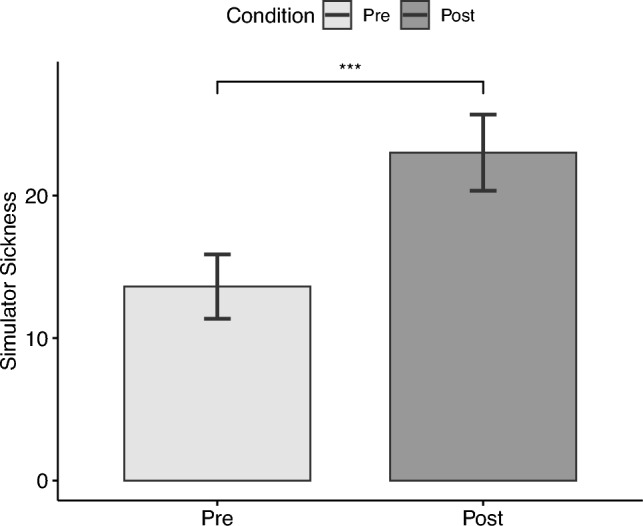
Simulator sickness.

In order to search for outliers in the behavioral markers of working memory capacity and creativity, we performed Grubbs test on the difference score of each measure (i.e., DSB and AUT). This score was calculated by subtracting the respective value of the no-plant condition from the values of the plant condition. No outliers could be found using this method. According to Shapiro-Wilk tests, some data were normally distributed (DSB-mean span, AUT, PANAS-positive affect and ZIPERS-positive affect) and some were not (DSB-maximum length, PANAS-negative affect, ZIPERS sub-scales Fear Arousal, Anger and Aggression, Attentive Coping , and Sadness as well as PRS, Sense of presence, SSQ). To avoid switching between statistical tests, we decided to report our analysis based on parametric tests, as paired t-tests have been shown to be robust against deviations from normality^62,63^. The significance level was set at .05. As a measure of effect size, we used Cohen’s d, which is commonly classified into small ($$|d| =.2$$), medium ($$|d| = .5$$), and large ($$|d| = .8$$) effects^64,65^. The main results are plotted in Figs. 2, 3, 4, 5, 6, 7 and 8, where asterisks represent *p* values (*$$p <.05$$, **$$p <.01$$, ***$$p <.001)$$.

### Digit span backward test (DSB)

The mean span (MS) was significantly ($t(38)=2.34, p=.02, |d|=.39$) higher for the plants condition ($M=5.71, \textit{SD}=1$) compared to the no-plants condition ($M=5.39,\textit{SD}=.87$) which supports H1.a. No significant differences ($t(38)=1.75, p=.09, |d|=.29$) in the given maximum length of the responses could be observed between the plants ($M=5.92, \textit{SD}=1.02$) and no-plants ($M=5.69, \textit{SD}=.92$) conditions (see Fig. 2).

### Alternative uses task (AUT)

The average creativity ratings (see Fig. 3) were significantly higher ($t(38)=2.06,p=.047,|d|=.35$) for the condition with plants ($M=5.95,\textit{SD}=1.75$) than the no-plants condition ($M=5.55,\textit{SD}=1.82$). This finding confirms H1.b and with both parts of the first hypothesis (H1.a and H1.b) being supported, H1 can be confirmed. This means that the presence of virtual plants lead to higher convergent and divergent cognitive performance in the virtual office environment.

### Positive and negative affect schedule (PANAS)

We observed significantly higher values ($t(38)=3.78, p=.0005$) for positive affect (see Fig. 5) after exposure to the plants condition ($M=3.25, {SD}=.68$), with a moderate effect size ($|d| =.61$) compared to the no-plants condition ($M=2.93, {SD}=.62$). No significant differences ($t(38)=-1.71, p=.095, |d|=.27$) could be observed for negative affect, which still showed slightly lower values after the plants condition ($M=1.27, {SD}=.37$) compared to the no-plants condition ($M=1.36, {SD}=.37$).

### Zuckerman inventory of personal reactions (ZIPERS)

The level of Fear Arousal sub-scale did not differ significantly ($t(38)=.27, p=.79, |d|=.04$) between the plants ($M=1.44,{SD}=.44$) and no-plants ($M=1.47,{SD}=.54$) conditions. However, similar to the effect measured by PANAS, the Positive Affect measured by ZIPERS (see Fig. 4) showed significantly higher values ($t(38)=-3.92, p=.0003,|d|=.63$) after the plants condition ($M=2.97,{SD}=.81$) compared to the no-plants condition ($M=2.54,{SD}=.77$). The values for the Anger and Aggression sub-scale were also significantly lower ($t(38)=2.25, p=.03, |d|=.36$) after the plants condition ($M=1.24,{SD}=.48$) than after the no-plants condition ($M=1.44,{SD}=.49$). Additionally, Attentive Coping was significantly higher ($t(38)=-3.1, p=.003, |d|=.51$) after the plants condition ($M=3.71,{SD}=.81$) compared to the no-plants condition ($M=3.21,{SD}=.99$). Sadness did not significantly differ ($t(38)=.7, p=.49, |d|=.11$) between the plants ($M=1.05,{SD}=.22$) and no-plants conditions ($M=1.1,{SD}=.38$).

Taking the results of both PANAS and ZIPERS into account, we can conclude that H2 is partially supported. This indicates that some measures of psychological well-being show better values, i.e., higher positive affect and attentive coping as well as lower anger and aggression, as a result of an exposure to a virtual office environment with virtual plants through VR. Other measures showed no significant differences.

### Perceived restorativeness scale (PRS)

The total perceived restorativeness (see Fig. 6) for the plants condition ($M=4.99,{SD}=.82$) was significantly higher ($t(38)=-5.82, p<.001, |d|=.93$) than the no-plants condition ($M=4.37,{SD}=.79$), which supports H3.

### Sense of presence

The sense of presence (see Fig. 7) in the virtual environment was significantly higher ($t(38)=-2.66, p=.01, |d|=.43$) for the condition with plants ($M=5.59,\textit{SD}=.82$) compared to the no-plants condition ($M=5.23,\textit{SD}=.87$), confirming H4.

### Simulator sickness questionnaire (SSQ)

There was a significant increase ($t(38)=4.42, p<.001, |d|=.71$) of the total simulator sickness values from the pre- ($M=13.62,\textit{SD}=14.09$) to post-exposure measurements ($M=23.02,\textit{SD}=16.67$). This signifies that the entire experiment increased the symptoms of simulator sickness (see Fig. 8) .

### Self-reports of performance and relationship with real plants

At the end of the study, we asked participants to compare their experience in both environments and tell us in which virtual environment they thought they performed better. As a result, 51% of them (N = 20) rated their performance in the virtual office with plants condition better, 18% (N = 7) thought they performed better in the office with no plants, and the rest (31%, N = 12) had the impression that their performance in both environments was similar. We also asked them which environment they liked more. The majority (82%, N = 32 ) liked the plant condition more, 18% (N = 7) liked both environments similarly, and no one liked the no-plants condition more than the office with plants.

In addition, we asked some questions about the presence of real plants in participants’ everyday life. The results revealed that only two participants (5%) did not have any plants at home. The rest (95%) have a range of a few to a large number of plants in their homes. At their workplaces, more than half of the participants (54%, N = 21) had plants, while 26% (N = 10) were considering getting some, and 20% (N = 8) had no plants at work and were not considering getting any. Finally, 38% of the participants (N = 15) believed that plants have some influence on their mood and cognitive performance while 41% (N = 16) thought this influence is large and 21% (N = 8) assumed that this influence is actually very large.

## Discussion

### Findings

In this paper, we examined the effects of virtual plants in an office environment experienced in VR on the user’s cognitive performance and psychological well-being. In contrast to previous work in this area^19,40,41^, we examined this independent variable in isolation (i.e., the only difference between the two environments were the added plants) which ensured that the observed effects cannot be attributed to other confounding variables. Based on this design, we formulated four hypotheses.

First, we hypothesized that exposure to our virtual office environment with virtual plants, compared to an exposure to the same virtual environment without plants, leads to higher cognitive performance. We considered both convergent and divergent cognitive functioning and therefore administered both a working memory task as well as a creativity task. As a working memory task, we used the digit span backward test and measured the maximum length and mean span of correct responses. The results of the mean span (but not the maximum length) of the digit span backward test as well as the creativity task support the hypothesis, confirming findings from previous research on real nature exposure^20^, and generalizes these findings to a virtual context.

Our findings match the results of several previous studies that have shown better performance in a digit span backward test after nature exposure^66–72^. Also, our findings extend previous research on biophilic indoor environments. For instance, Yin et al.^40^ observed significantly better performance in a digit span backward test for their real biophilic indoor environment compared to the non-biophilic one. However, they observed no significant differences between the cognitive performance in virtual representations (i.e., $360^\circ$ videos) of the same environments in VR. The differences between the type of presentation of the cognitive test to the participant (i.e., visual in their study vs. auditory in our study) as well as the virtual environment (i.e., $360^\circ$ videos vs. computer-simulated virtual environments) might have contributed to this difference in observations.

In addition, we observed better performance in our creativity task in the presence of virtual plants in the virtual office environment. This finding is also in accordance with previous research which demonstrated that creativity can benefit from exposure to nature. For example, one study observed an increase of creative performance when there were plants in the (physical) room^39^. Similar to real nature exposure, exposure to computer-generated biophilic office designs in VR showed in our study also positive effects on creativity.

Thus, our results confirm that the presence of virtual plants leads to higher convergent and divergent cognitive performance in a virtual office environment. Several studies have suggested that the reason for the improved cognitive performance lies in the restoration of directed attention according to the attention restoration theory (ART). Tasks such as the digit span backward test, which are associated with high cognitive demand, are therefore particularly sensitive to restored attention^73^. In our study, we also assessed the four components of the ART through a dedicated questionnaire, the perceived restorativeness scale (i.e., PRS). Our results revealed higher perceived restorativeness values for our biophilic virtual office environment compared to our non-biophilic office environment. Therefore, a reason for cognitively better performance at the presented tests could be the restorativeness qualities of the virtual plants.

The results of our psychological well-being measures, namely PANAS and ZIPERS, support that experiencing the plants condition increases positive affect and attentive coping while decreasing feelings of anger and aggression compared to the no-plants condition. Psychological well-being measures show better values after VR exposure in some dimensions (e.g., positive affect) while other measures (i.e., sadness, fear arousal, negative affect) showed no significant differences. These results are in line with a large body of research, in which the positive effects of exposure to nature on psychological well-being have been demonstrated in both physical and virtual environments^9,12,42,66,67,74–80^. These results could be explained by the stress reduction theory (SRT) which suggests that contact with nature leads to a reduction in stress, which in turn leads to an improvement in positive emotions and a reduction in stress-related feelings.

Finally, the presence of virtual plants led to a higher sense of presence in the virtual office environment, which is another novel finding of our present study. Unfortunately, we did not measure simulator sickness after each condition and instead administered it only at the start, i.e., before the first VR exposure, and after the second VR exposure, i.e., at the end of the experiment. With this, we observed that the entire experiment somewhat increased the symptoms of simulator sickness, but at a generally low level. The total value of the simulator sickness questionnaire can vary from 0 to 235.62^61^. On this scale, the observed simulator sickness remained at an overall low level, with M = 13.62 before exposure and M = 23.02 after exposure to both conditions in VR. A reason for this could be that the participants were seated at a single stable location in the virtual environment, which reduced the overall sensory conflict(s) known to contribute to motion sickness^59,60,81^. Also, participants filled out the SSQ outside of VR and on a separate computer. The restoring effect of the real environment could have contributed to lower SSQ values as well.

Given that plants are known to be positive for work environments^37,38^, our evidence strongly supports their use in future immersive working and learning environments, which could then have positive effects on performance or productivity. But this clearly needs to be verified in future studies.

### Limitations and future work

Although the findings of our study are in line with or extend previous work and provide interesting implications for the design of future immersive working and learning environments, we acknowledge some potential limitations of our experimental design.

In our study design, the participants’ psychological well-being was measured solely through questionnaires that were presented after VR exposure. Future studies may consider inclusion of physiological measures such as heart rate or skin conductance that can reveal participants’ psycho-physiological responses during the VR exposure.

Another limitation of the current study is the lack of qualitative analysis of well-being. Although we asked about the presence of real plants in participants’ living and working spaces, we could not be sure about their position towards the effects of virtual plants on their well-being beyond what we could measure through our standardized tests and questionnaires. We only know that they liked the environment with plants more and they thought their performance was better in that environment. Future studies may consider including qualitative instruments to capture more of what standard questionnaires may not explicitly measure.

The comparison of an office that is “fuller” with plants with a “less full” office without plants also represents a potential limitation of our study. Thus, we cannot completely rule out that the observed effects could be attributed to that difference. An alternative would be to place other (non-nature) items into the environment instead of the plants. Yet, we decided against this in the present study, since in this case plants would also have been (at least indirectly) compared with other objects, which might have attracted different forms of visual interest to these objects. Consequently, it might be useful to use eye tracking to assess the participants’ actual gaze directions in future work, to assess if they actually directly attend to the virtual plants or other objects.

The VR experience in this study was also limited to the modality of sight. Future studies could enhance the immersion by including other senses such as sound, smell, and touch. Also, users were not embodied in our virtual environment. Granting a virtual body to users in future studies could potentially enhance the sense of presence in the virtual world.

Finally, another limitation of the present study is that the reported effects were measured after one-time exposure. Therefore, no statement can be made about repeated exposures or long-term effects of virtual biophilic office environments. Furthermore, with a fairly young sample in our study with an average age of 24.15 years and from a WEIRD (western, educated, industrialized, rich, and democratic) society^82^, the findings of this study cannot be generalized to other groups of users. Therefore, future studies may consider repeating this study with a different population, such as older adults and in different contexts, to investigate how the current findings might or might not generalize to more diverse and larger participant groups, different tasks, and realistic work scenarios. Bringing together the individual studies could then provide information as to whether and under what conditions VR can simulate the benefits of nature, so that people living in urbanized environments can benefit more from the power of nature.

## Conclusion

The aim of this study was to investigate the effects of virtual biophilic office design on the user’s cognitive performance and psychological well-being in immersive systems. We thus conducted a VR experiment, where participants experienced a virtual office environment either in the presence or absence of virtual plants. In contrast to previous work in this area, we only examined this independent variable in isolation, i.e., the only difference between the two environments were the added plants, which ensured that the observed effects cannot be attributed to other influencing variables.

In summary, our results provide clear support for the use of biophilic design in virtual reality environments. We demonstrated that the inclusion of 3D models of natural elements, in particular plants, in a virtual office environment results in better convergent and divergent cognitive functioning inside VR and better psychological well-being after VR exposure. Also, the participants perceived the plant condition as more restorative and felt significantly more present in the virtual environment with virtual plants.

These results provide important implications for the design of future VR environments, in particular in the area of new working and learning environments or research on human-building interaction^83^. Thus, to benefit the most from the positive effects of virtual nature, future VR experiences should consider corresponding designs of their virtual environments.

## Supplementary Information

Supplementary Information.

## Data Availability

The datasets used and analysed during the current study will be available from the corresponding author on reasonable request.
